# Promoter tools for further development of *Aspergillus oryzae* as a platform for fungal secondary metabolite production

**DOI:** 10.1186/s40694-020-00093-1

**Published:** 2020-03-23

**Authors:** Maiko Umemura, Kaoru Kuriiwa, Linh Viet Dao, Tetsuya Okuda, Goro Terai

**Affiliations:** 1 Bioproduction Research Institute, National Institute of Advanced Industrial Science and Technology (AIST), Ibaraki, 305-8566 Japan; 2 Computational Bio Big Data Open Innovation Laboratory, AIST, Ibaraki, 305-8566 Japan; 3 Department of Zoology, National Museum of Nature and Science, Ibaraki, 305-0005 Japan; 4 Department of Computational Biology and Medical Sciences, Graduate School of Frontier Sciences, University of Tokyo, Chiba, 277-8561 Japan; 5 Present Address: Department of Biomedical Engineering, Faculty of Engineering, National University of Singapore, 4 Engineering Drive 3, Singapore, 117583 Singapore

**Keywords:** *Aspergillus oryzae*, Promoter, Secondary metabolite production

## Abstract

**Background:**

The filamentous fungus *Aspergillus oryzae* is widely used for secondary metabolite production by heterologous expression; thus, a wide variety of promoter tools is necessary to broaden the application of this species. Here we built a procedure to survey *A. flavus* genes constitutively highly expressed in 83 transcriptome datasets obtained under various conditions affecting secondary metabolite production, to find promoters useful for heterologous expression of genes in *A. oryzae*.

**Results:**

To test the ability of the promoters of the top 6 genes to induce production of a fungal secondary metabolite, ustiloxin B, we inserted the promoters before the start codon of *ustR*, which encodes the transcription factor of the gene cluster responsible for ustiloxin B biosynthesis, in *A. oryzae*. Four of the 6 promoters induced ustiloxin B production in all tested media (solid maize, liquid V8 and PDB media), and also *ustR* expression. Two of the 4 promoters were those of *tef1* and *gpdA*, which are well characterized in *A. oryzae* and *A. nidulans*, respectively, whereas the other two, those of AFLA_030930 and AFLA_113120, are newly reported here and show activities comparable to that of the *gpdA* promoter with respect to induction of gene expression and ustiloxin B production.

**Conclusion:**

We newly reported two sequences as promoter tools for secondary metabolite production in *A. oryzae*. Our results demonstrate that our simple strategy of surveying for constitutively highly expressed genes in large-scale transcriptome datasets is useful for finding promoter sequences that can be used as heterologous expression tools in *A. oryzae*.

## Background

The filamentous fungus *Aspergillus oryzae* has been traditionally used in Japanese fermentation industries to produce sake, shoyu and miso, as well as in enzyme production industries. *A. oryzae* has also been used as a host for production of fungal secondary metabolites, e.g., cyclopiazonic acid [1] and 1,3,6,8-tetrahydroxynaphthalene [2], mainly because *A. oryzae* scarcely produces secondary metabolites that could otherwise confound the isolation of target compounds [3]. Many genetic tools have been developed for *Aspergillus oryzae*; *e.g.*, constitutive and inducible promoters as described below, auxotrophic (*pyrG* [4], *argB* [5], *niaD* [6], *sC* [7] and *adeA* [8]) and dominant (*amdS* [9] and *ptrA* [10]) selective markers, a marker recycling system [11], a quadruple auxotrophic transformation system [12], and genome editing systems [13, 14]. These tools facilitate simultaneous integration of several genes into the fungal genome, which is necessary for heterologous production of fungal secondary metabolites because usually several genes are involved in their biosynthesis. *A. oryzae* NSAR1, the quadruple auxotrophic strain (*argB*^*−*^, *niaD*^*−*^, *sC*^*−*^ and *adeA*^*−*^) [12], is used to produce fungal secondary metabolites by simultaneously introducing two to nine genes for biosynthesis of such compounds as pleuromutilin [15], paxilline [16], terretonin [17], helvolic acid [18], menisporopsin A [19] and asperipin-2a [20]. A variety of basidiomycete terpenes have been successfully produced in *A. oryzae* by heterologous expression of their respective biosynthetic genes using the genome-editing system [21].

Although the number of promoters that can be used for heterologous expression in filamentous fungi is limited in comparison with that in the yeast *Saccharomyces cerevisiae*, where a well-established set of promoters covers virtually all patterns of expression [22], promoter tools have been developed for filamentous fungi including *Trichoderma reesei* [23], *A. niger* [24], *Penicillium chrysogenum* [25] and *Ustilago maydis* [26]. In *A. oryzae*, the maltose-inducible promoter of the Taka-amylase A gene (*amyB*) [27–29] is often used for heterologous expression [16, 20, 30], as are the thiamine-inducible promoter of the thiamine thiazole synthase gene (*thiA*) [31] and the constitutive promoter of the translation-elongation factor 1α (*tef1*) [32]. The *glaA* promoter of the glucoamylase A gene was originally characterized in *A. niger* [33], and was then also used in *A. oryzae* for secondary metabolite production and gene functional analyses [28, 34, 35]. The native *A. oryzae* promoter of the oxidoreductase gene, *kojA*, which is involved in kojic acid biosynthesis successfully induced the expression of the polyketide synthase gene (*wA*) and production of the respective polyketide, YWA1 [36]. Whilst mainly native promoters are used for heterologous expression in filamentous fungi [37], in *S. cerevisiae*, universal expression systems for fungal genes comprising a set of synthetic promoters and transcription factors have been recently developed to synthesize a wide range of fungal natural products [38–40]. However, because *A. oryzae* possesses a variety of proteins and secretion systems for proteins and low-molecular-weight compounds that differ from those in *S. cerevisiae* [41–43], finding additional promoters that would be functional in *A. oryzae* is important for the use of this species as a heterologous expression host in addition to *S. cerevisiae*.

Whilst *A. oryzae* almost never produces secondary metabolites except kojic acid [44], *A. flavus* produces quite a few secondary metabolites, including aflatoxin, a strong carcinogen, and cyclopiazonic acid, which is toxic in large amounts. *A. oryzae* was once proposed to be reduced to an *A. flavus* subspecies because of its 100% DNA complementarity with *A. flavus* [45], but was retained as a separate species due to economic and food safety concerns [46]. Georgianna et al. [47] investigated the transcriptomic pattern of *A. flavus* NRRL3357 under 28 different conditions affecting secondary metabolite production. They classified the 55 putative secondary metabolite biosynthesis (SMB) genes encoding polyketide synthases, non-ribosomal peptide synthetases and terpene synthases into four clades according to their expression patterns, and found that the SMB genes in the two clades are expressed at lower levels in *A. oryzae* than in *A. flavus* [47]. Ehrlich et al. [48] reported that some putative SMB genes in *A. flavus* are absent or expressed at significantly lower levels than in *A. oryzae*. These reports indicate that the genes necessary to produce secondary metabolites are likely to be expressed in *A. flavus* rather than *A. oryzae*, and thus *A. flavus* is likely to be suitable as a potential source of usable promoters that will activate secondary metabolism genes in *A. oryzae*.

Transcriptome datasets are used to identify constitutive promoters in bacteria [49] and plants [50]. Oda et al. [51] used transcriptome datasets collected under three different conditions to find sorbitol-inducible promoters in *A. oryzae*. Sibthorp et al. [52] used transcriptome datasets obtained under five different culture conditions for the global identification of promoters in *A. nidulans*. Here we extracted information about constitutively highly expressed *A. flavus* genes by analyzing the 75 publicly available [47] and 8 newly obtained transcriptome datasets generated under 32 conditions affecting secondary metabolite production. To the best of our knowledge, no such large-scale dataset analysis has been used to find constitutive promoters in filamentous fungi, at least in *A. oryzae* or *A. flavus*. We examined whether the promoter sequences of the 6 prioritized genes would enhance downstream gene transcription and secondary metabolite production in *A. oryzae* by inserting them just before the start codon of *A. oryzae ustR*, the gene encoding the transcription factor of the ustiloxin B biosynthetic gene cluster [34]. Ustiloxin B, a fungal secondary metabolite, is a toxic cyclic peptide originally isolated from the plant pathogenic fungus *Ustilaginoidea virens* [53, 54]. The biosynthetic gene cluster for ustiloxin B has been identified in *A. flavus*, revealing that ustiloxin B belongs to a relatively new class of fungal secondary metabolites, ribosomally synthesized and post-translationally modified peptides (RiPPs) [34]. *A. oryzae* does not produce the compound but possesses a gene cluster identical to that in *A. flavus* except the lack of an approximately 2 kb upstream region of *ustR* [34]. When the lacking promoter region of *ustR* is compensated with the *glaA* promoter, *A. oryzae* starts to produce ustiloxin B [35]. Therefore, we can efficiently assess the activity of a sequence as a promoter from ustiloxin B production by an *A. oryzae* transformant in which the target sequence is inserted before *ustR*.

## Results and discussion

**Fig. 1:**
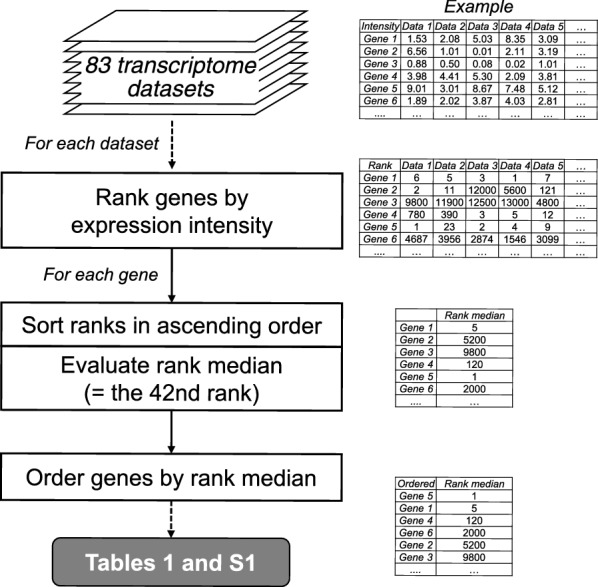
The procedure for computational prioritization of *A. flavus* genes from 83 transcriptome datasets. Processes are shown in rectangles with corners

We ranked the 13,481 genes of *A. flavus* by the median of their expression ranks among the 83 (8 in-house and 75 publicly available) transcriptome datasets to identify constitutively highly expressed genes (Fig. 1, Additional file 1: Table S1). We included the in-house data generated under the conditions where ustiloxin B was produced, because ustiloxin B production by *A. flavus* and the corresponding biosynthetic pathway were not known when the publicly available datasets were published. To obtain the in-house data, we cultured the *A. flavus ustR*^*OE*^ and control strains in V8 vegetable juice (V8) or potato dextrose broth (PDB) liquid medium, where the strains produced ustiloxin B [34]. The publicly available data were obtained under 28 different conditions affecting secondary metabolite production, such as maize and wheat culture, for *A. flavus* NRRL3357 and the deletion and overexpression mutants of *laeA*, a global secondary metabolism regulator of gene expression [55], with *A. oryzae* RIB40 used as a control [47].

We selected the top 6 genes for *ustR* expression test using their 5′-untranslated region (UTR) sequences as promoters (P1–P6; Table 1). P1, P2 and P5 have been reported as promoter tools for robust transcription of downstream genes in *A. oryzae* (P1 [32] and P5 [56]) or *A. nidulans* (P2 [57, 58]). The gene containing P4 (AFLA_113120) is indispensable in *A. flavus* for normal fungal growth and development, aflatoxin biosynthesis and seed colonization [59], but the promoter sequence has not been tested as a gene expression tool. P3 drives AFLA_014570, which is annotated to encode a conserved hypothetical protein.

**Table 1:** Top 13 genes constitutively highly expressed in 83 transcriptome datasets

Promoter name (for tested 5′-UTRs)^a^	Gene	Expression rank			Description in NCBI	5′-UTR size to next gene (bp) (size of tested sequence)	References
		Minimum	Maximum	Median			
P1	AFLA_090780	1	32	5	Translation elongation factor EF-1 alpha subunit (TEF1)	1309 (1029)	Kitamoto et al. [32]
–	AFLA_064900	1	9772	12	Hypothetical protein	525	
P2	AFLA_025100	3	886	12	Glyceraldehyde 3-phosphate dehydrogenase (GpdA)	1792 (1024)	Punt et al. [57]
P3	AFLA_014570	1	313	12	Conserved hypothetical protein	3461 (1024)	This study
–	AFLA_050690	3	174	16	Mitochondrial ADP/ATP carrier protein	314	
P4	AFLA_113120	5	177	18	GPI-anchored cell wall organization protein (Ecm33)	6093 (1024)	This study
–	AFLA_048690	1	1022	18	Alcohol dehydrogenase, putative	2918	
–	AFLA_063260	1	11,577	19	Conserved hypothetical protein	409	
–	AFLA_083050	1	98	21	40S ribosomal protein S25	345	
P5	AFLA_052860	1	555	27	Chaperone/heat shock protein (Hsp12)	967 (967)	Koda et al. [56]
–	AFLA_047870	7	128	34	40S ribosomal protein S1	641	
–	AFLA_006300	8	570	34	Nucleoside diphosphate kinase	2864	
P6	AFLA_030930	6	140	34	Fructose-bisphosphate aldolase, class II	2864 (1024)	This study

^a^Genes with 5′-UTRs shorter than 950 bp and those with maximum expression ranks larger than 1000 were omitted

**Fig. 2:**
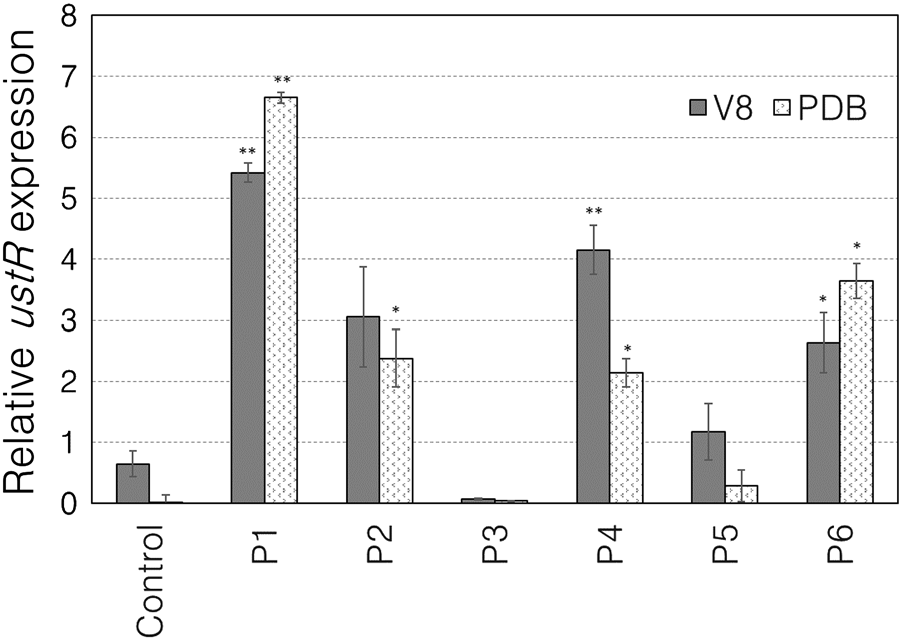
Relative *ustR* transcript levels (normalized to those of the tubulin transcript) in *A. oryzae* transformants with promoters P1 to P6 fused to *ustR* after 3-day culture in liquid PDB and V8 media. The error bars represent the standard errors of the three replicates in a sample. *p < 0.05, **p < 0.01 by paired *t*-test against the control *pyrG* revertant

To assess the degree of gene transcription activity induced by the promoters, we measured the *ustR* expression levels (relative to those of the tubulin transcript) in the transformants cultured in V8 and PDB liquid media. The relative expression level of *ustR* was highest in the P1 transformant, followed by those in the P4, P2, P6 (V8) or P6, P2, P4 (PDB) and P5 transformants (Fig. 2). P1 is the well-characterized promoter of *tef1* [32] and P2 is that of *gpdA* reported in *A. nidulans* [57, 58]; accordingly, the P1 and P2 transformants showed respective ≈six- and ≈three-fold relative expression levels of *ustR* against tubulin. The P3 transformant showed negligible relative expression levels of *ustR* in both V8 and PDB media. The P5 transformant showed ≈1- and ≈0.3-fold relative expression levels of *ustR* against tubulin in V8 and PDB media, respectively, which are the smallest levels next to P3. P5 reportedly increase the relative mRNA abundance of a β-glucuronidase (GUS) gene from *Escherichia coli* in comparison with that of 18S rRNA to ≈1.4 at 30 °C in DP medium (2% dextrin, 1% polypeptone, 0.5% KH_2_PO_4_, and 0.05% MgSO_4_·7H_2_O) in *A. oryzae* [56]. Because the media and the standard genes are different, it is difficult to compare the induction efficiency of P5 between the current and previous studies.

**Fig. 3:**
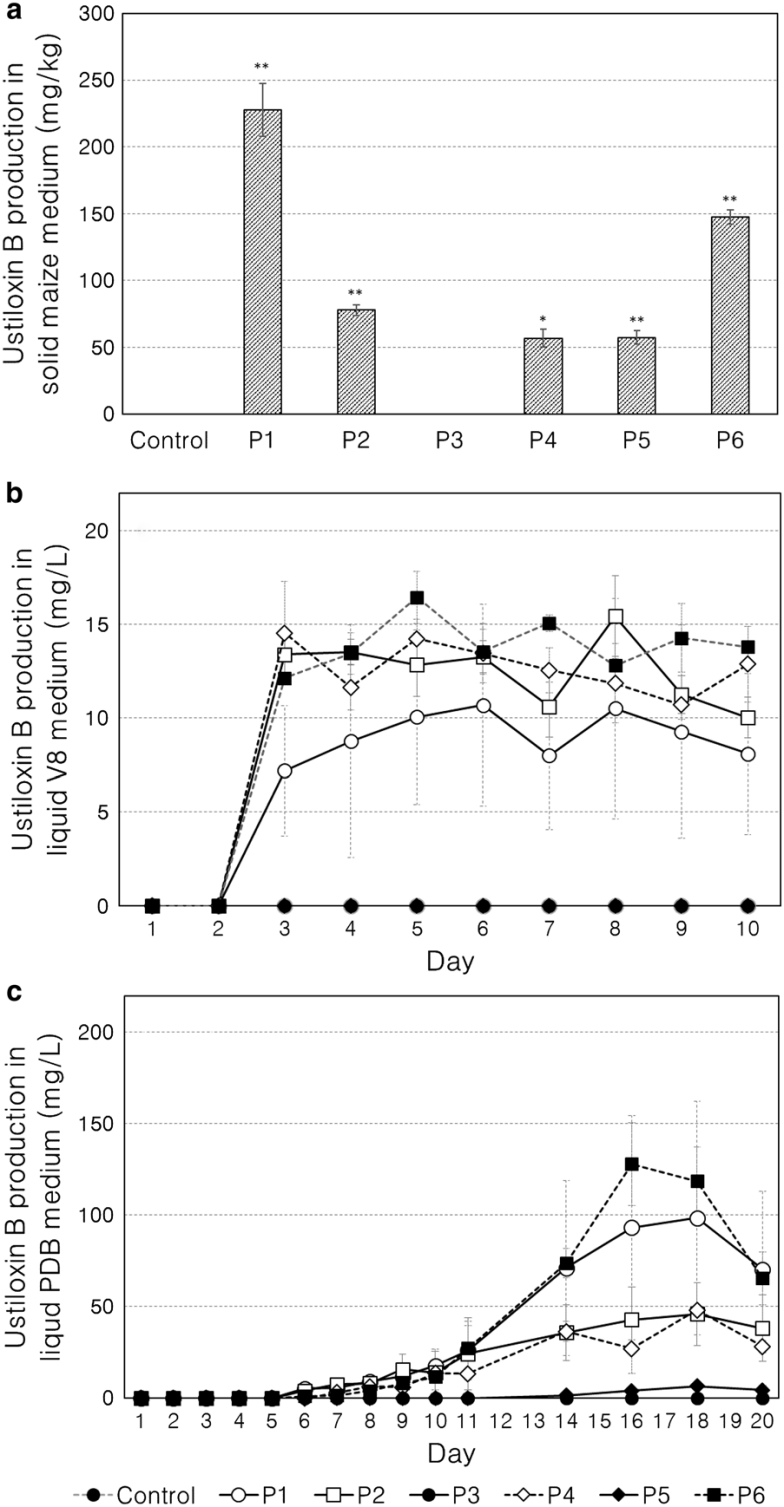
Ustiloxin B production by *A. oryzae* transformants with promoters P1 to P6 fused to *ustR* in (**a**) solid maize medium for 14 days, **b** V8 liquid medium for up to 10 days, and **c** PDB liquid medium for up to 20 days. The error bars represent the standard errors of the three replicates. The error bars of P1 are drawn in dashed line in **b**, **c** to distinguish them from others. In **a**, *p < 0.05, **p < 0.01 by paired *t*-test against the control *pyrG* revertant

We tested ustiloxin B productivity by the transformants under three different conditions, *i.e.*, solid cracked maize and liquid V8 and PDB media. Ustiloxin B production by *A. flavus* was also confirmed in solid cracked maize [34]. Ustiloxin B was produced by the transformants with the tested promoters except P3 in the cracked maize solid culture (Fig. 3a). The largest yield was > 220 mg/kg in the P1 transformant, followed by the transformants with P6, P2, P5 and P4; the latter two transformants had identical yields.

In V8 liquid culture, ustiloxin B started to be produced on the 3rd day by the transformants with P1, P2, P4 and P6, but the yield did not increase after that (Fig. 3b). The yield tended to be unstable, with large differences among replicates. Unlike in the maize culture, the P1 transformant had the lowest yield (ca. 10 mg/L), whereas the yields were similar among the transformants with P2, P4 and P6 (ca. 15 mg/L). The P5 transformant did not produce ustiloxin B in liquid V8 culture, unlike in solid maize culture. The transformant with P3 did not produce ustiloxin B at all, either in V8 liquid culture or in maize culture.

In PDB liquid culture, ustiloxin B started to be produced by the P1, P2, P4 and P6 transformants on the 6th day, which was 3 days later than in V8 liquid culture. However, the transformants kept producing ustiloxin B up to around the 18th day; the highest yield (almost 120 mg/L) was achieved by the P6 transformant, followed by that with P1 and by those with P2 and P4. The transformant with P5 started to produce the compound on the 14th day, but the maximum yield was only around 6 mg/L at the 18th day, much lower than those achieved by the other four transformants. The P3 transformant did not produce ustiloxin B at all.

The order of the ustiloxin B production in PDB liquid medium was concordant with that of relative *ustR* expression in PDB liquid medium (Fig. 3c), as well as in solid maize culture except that the P4 and P5 transformants showed nearly identical ustiloxin B production (Fig. 3a). In the V8 medium, the ustiloxin B production by the P1 transformant was the lowest among those of the P1, P2, P4 and P6 transformants, even though the P1 transformant showed the highest relative *ustR* expression in the V8 medium. No ustiloxin B production by the P3 transformant was observed in any of the three media tested, in accordance with the almost 0 relative *ustR* expression in the P3 transformant. Total fungal cell weight did not differ among transformants cultured in either of the liquid media and was on average around 150 mg in V8 and 1.2 g in PDB cultures (Figure S2). We did not measure ustiloxin B yield or cell weight at 20 days in V8 liquid medium because the yield plateaued in 10 days; the slow production of the compound in PDB medium might have allowed high yield.

In summary, our results show that 4 promoters (P1, P2, P4 and P6) among 6 selected by our survey of 83 transcriptome datasets worked well to enhance the transcription of the key gene for fungal secondary metabolite production and the production of such a compound. P1 and P2 have been already reported and are widely used as constitutive promoters [32, 57]. P1 or the *tef1* promoter reportedly induces to produce *S. cerevisiae* proteins (41 kDa) at ≈ 100 mg/L in glucose medium [32], which yield is comparable to our result of ustiloxin B (Mw 645.2) production at ≈ 100 mg/L in PDB liquid medium, 18 days (Fig. 3c). P2 or the *gpdA* promoter reportedly induces the expression of the endogenous gene, *amdS*, encoding acetamidase up to 30-fold in *A. nidulans* [58], whereas our result showed ≈four-fold relative expression level of *ustR* against tubulin in V8 medium (Fig. 2). In the 83 datasets used in this study, the average gene expression value of the β-tubulin gene (AFLA_051840) is 11.5, whereas that of an acetamidase gene homologous to *A. nidulans amdS* (AFLA_036780) is 6.6. The β-tubulin gene showed the 1.7-fold relative expression level against the *amdS*-homolog gene, suggesting that the gene induction activity of P2 was at the one-quarter weaker level in our study than in the previous report. P4 and P6 were newly identified in this study and showed useful activity in terms of both gene induction and secondary metabolite production. Their activities were comparable to those of P2, the well-characterized promoter of *gpdA* in *A. nidulans* [57, 58]. The P4 and P6 sequences were listed in Table S2. P3 did not induce either gene expression or compound production. The gene corresponding to P3 (AFLA_014570) is annotated as a “conserved hypothetical protein”; thus, a more informative annotation might require investigation of its coding sequence and other elements. The P5 promoter showed low gene transcription activity especially in PDB medium, resulting in no or scarce ustiloxin B production in V8 or PDB liquid media, contrary to a previous report that P5 greatly enhanced the transcription and translation efficiency of GUS mRNA in *A. oryzae* [56]. The P5 promoter comes from a gene (AFLA_052860) for a chaperon or heat shock protein; P5 might not be suitable for the culture conditions at 30 °C used for secondary metabolite production.

We combined the publicly available datasets (GSE15435) with in-house datasets (GSE136041) to prioritize the constitutively highly expressed genes. By using large-scale analysis of 83 transcriptome datasets obtained under 32 different culture conditions, we were able to stabilize the prioritized gene list (Additional file 1: Table S3). When we used only GSE136041, which was obtained under 4 conditions where ustiloxin B was produced, only the *tef1* promoter was selected among the tested 6 promoters, and neither of the two new promoters (P4 and P6) was detected.

We did not test the 5′-UTR sequences shorter than 950 bp, even if the corresponding genes were constitutively highly expressed according to our survey. As our results have validated our surveying strategy for finding useful promoters for heterologous expression in *A. oryzae*, these shorter 5′-UTR sequences might be also worth testing, as short promoters make the construct small and convenient for transformation.

## Conclusion

In this study, we showed that 4 promoters (P1, P2, P4 and P6) out of the 6 tested are suitable to enhance gene transcription for fungal secondary metabolite production in *A. oryzae*. To the best of our knowledge, P4 and P6 (5′-UTRs of AFLA_113120 and AFLA_030930, respectively) have not been previously reported as useful promoters. The performances of P4 and P6 in induction of the expression of a downstream gene and ustiloxin B production were comparable to those of P2, which is an well-characterized constitutive promoter of *gpdA* in *A. nidulans* [57, 58]. The identification of P4 and P6 shows that our simple ranking strategy using large sets of transcriptome data obtained under conditions affecting secondary metabolite production was able to prioritize genes whose promoter regions can be useful for enhancing translation of genes of interest under certain conditions in *A. oryzae*.

## Methods

### Fungal strains

*Aspergillus oryzae* NS4DLDP (RIB40 *ΔligD::ptrA niaD*^*−*^* ΔpyrG::sC* of *A. nidulans*) [44] was used as the parental strain to construct the transformants in which the selected 6 different promoter sequences were inserted before *ustR* (NCBI Gene ID 5,995,877).

*A. flavus ustR*^*OE*^ strain along with the *pyrG* marker revertant as a control, which were previously constructed from the *A. flavus* CA14 *Δku70 ΔpyrG* strain [34], were used for the microarray assay as described below. In the *ustR*^*OE*^ strain, the constitutive *tef1* promoter was inserted before the start codon of *ustR* (composed of NCBI Gene IDs 7917921 and 7917922).

The genome information with gene annotations of *A. flavus* NRRL3357 (NCBI acc. nos. EQ963472.1–EQ963493.1) was applied to genes of *A. flavus* CA14 derivatives and *A. oryzae* RIB40 used in the publicly available transcriptome data (GSE15435), as well as for the design of the microarray slide and primers.

### Microarray assay

DNA microarray assay was performed with a one-color method as described previously [60]. Briefly, 10^5^ conidia of *A. flavus ustR*^*OE*^ or the *pyrG* revertant strain were inoculated into 30 mL of V8 (20v/v% V8 juice [Campbell’s, Camden, NJ] containing 0.3w/v% CaCO_3_) or PDB (BD Biosciences, Franklin Lakes, NJ) liquid medium in a 100-mL flask and cultured for 2 days at 30 °C, 160 rpm. RNA was extracted from collected hyphae by using Isogen (Nippon Gene, Tokyo, Japan) and cDNA labeled with Cy3 was prepared by using a CyScribe cDNA Post-labeling Kit (GE Healthcare, Buckinghamshire, UK) according to the manufacturers’ instructions. The labeled cDNA mixture was hybridized at 42 °C for 15 h with a custom array slide designed for *A. flavus* on Agilent eArray (Agilent ID 052932; https://earray.chem.agilent.com/earray/) (Agilent, Santa Clara, CA) and, the slide was scanned on GenePix 4200A (Molecular Devices, San Jose, CA). The obtained data were normalized with the Agilent GeneSpring software. Transcriptome data obtained under four different conditions with two biological replicates for each, *i.e.*, 8 samples in total, was submitted as a series to NCBI Gene Expression Omnibus (acc. no. GSE136041) (https://www.ncbi.nlm.nih.gov/geo/). We added the 8 in-house datasets to the survey in this study because they were obtained under conditions where we confirmed the ustiloxin B production.

### Gene list

The GSE136041 microarray data and publicly available data obtained under 28 different conditions affecting secondary metabolite production (GSE15435, 75 sets in total) [47] were combined and used to prioritize the *A. flavus* genes constitutively highly expressed under conditions affecting secondary metabolite production conditions (Fig. 1). Genes were ranked according to their intensity values within each of the 83 sets and then re-ordered according to the median of the ranks among all 83 datasets (Additional file 1: Table S1). The median of the ranks of a gene was the 42^nd^ number in the list of 83 ranks sorted in ascending order. The genes whose maximum ranks among the 83 datasets were greater than 1000 were excluded because promoters of such genes were not likely to work constitutively by overviewing the list. The genes whose 5′-UTRs to the next upstream genes were shorter than 950 bp were also excluded, taking into account minimal regulatory spaces [61, 62]. The top 6 genes were then chosen for experimental examination (Table 1).

### Transformant construction

The selected 6 promoter sequences (each ≈1 kb; Table 1) were inserted upstream of *A. oryzae ustR* by homologous recombination using *pyrG* as the selective marker (Figure S1) as previously described [34]. Briefly, DNA constructs for transformation were prepared by concatenating the 1-kb 5′-UTR of *A. oryzae ustR*, *A. nidulans pyrG*, each selected promoter sequence, and 1 kb from the start codon of *A. oryzae ustR* via fusion PCR [63] using the primers listed in Additional file 1: Table S4. Approximately 1 µg of each DNA construct was transformed into *A. oryzae* NS4DLDP protoplasts using a PEG-mediated method. Three to five independent single colonies were screened by PCR amplification of the loci outside the *pyrG* marker and the candidate promoter sequence by using the primer pair 5′-TACTCCGTAAGTAATGCTCG-3′ and 5′-TGTCCGTCTTCATTACACTTC-3′.

### Metabolite analysis

Ustiloxin B was analyzed using liquid chromatography–tandem mass spectrometry (LC–MS/MS). The transformants and control *pyrG* revertant (1 × 10^5^ conidia each) were inoculated into 30 mL of V8 or PDB liquid medium supplemented with 70 mM (NH_4_)_2_SO_4_ in 100-mL flasks with a baffle and were incubated at 30 °C, 165 rpm rotation for 10 days for V8 cultures and 20 days for PDB cultures. Conidia were also inoculated in 50-mL glass vials each containing 2.5 g cracked maize kernels and 1.5 mL sterile water for 14 days. From V8 and PDB cultures, 100 µL supernatant was taken every 1 or 2 days and reacted with 200 µL ethyl acetate on a rotator for 2 h at room temperature. Solid maize cultures were extracted with 5 mL of 70% acetone, the acetone was evaporated, and then the residual water fraction was reacted with an equal amount of ethyl acetate for 2 h at room temperature on a rotator. After centrifugation at 21,130 × g for 10 min, 5 µL aliquots of the water phase were filtered through a 0.22-µm filter (P/N SLLGH04NL, Merck Millipore) and separated in a water–acetonitrile gradient (98:2 for 0.5 min and then a linear change to 20:80 for 3.5 min) at a flow rate of 0.4 mL/min on a 2.1 × 50 mm Acquity UPLC BEH C18 column, 1.7 µm (Waters, Milford, MA) in an LC–MS/MS system (Acquity UPLC H class and Xevo TQD, Waters). Three biological replicates were measured per sample. The ions of m/z 646 [M + H]^+^, expected for ustiloxin B (C_26_H_39_N_5_O_12_S, exact mass 645.23), were selected for MS/MS fragmentation, and the MS/MS chromatograms were analyzed to estimate the amounts of ustiloxin B from the peak areas at 2.0 min with the TargetLynx software (Waters).

### Quantitative PCR analyses of ***ustR***

Transformants with P1 to P6 were inoculated as for metabolite analysis (except that only PDB medium was used) and cultured at 30 °C, 165 rpm for 3 days. Approximately 50 mg of mycelia was collected and homogenized with 300 μL of zirconia beads (0.5 mm diameter) and 1 mL of Isogen II (Nippon Gene) at 7 m/s for 1 min twice with a 10 s interval on a Shakeman6 homogenizer (Biomedical Science, Tokyo, Japan). Total RNA was extracted according to the manufacturer’s instructions of Isogen II. Chromosomal DNA was removed from 10 μg total RNA by treatment with RNase-Free DNase I (New England Biolabs, Ipswich, MA), and the resulting samples were used as templates for cDNA synthesis using the PrimeScript II 1st strand cDNA Synthesis Kit (Takara Bio, Inc., Shiga, Japan). cDNA samples (2 μL; ≈6 ng/μL) were used for quantitative real-time PCR with a Kapa SYBR Fast qPCR Kit for Roche Light Cycler (Kapa Biosystems, Wilmington, MA) on a LightCycler 480 System II (Roche, Penzberg, Germany). Primers for *ustR* were 5′-cacagtcacctatatctacg-3′ and 5′-ggactgcatgttcttactt-3′, and those for the tubulin gene, used as an internal standard (NCBI Gene ID 5997350), were 5′-gaaactccacctccatcca-3′ and 5′-atctcgtccataccctcacc-3′. PCR conditions were initial incubation at 95 °C for 3 min followed by 40 cycles at 95 °C for 10 s, 60 °C for 20 s, and 72 °C for 1 s. The C_T_ values were evaluated using the second derivative maximum method with the instrument software for 3 biological replicates per sample, each with 3 technical replicates. The C_T_ values of the *ustR* and tubulin genes were converted to cDNA amounts according to the standard curves evaluated from serially diluted PCR amplicons using the above primers for each gene and genomic DNA of *A. oryzae* NS4DLDP as a template. The molar amount of *ustR* was normalized to that of tubulin for each replicate, and then averaged per sample.

## Supplementary information

**Additional file 1: Table S1.** Genes listed in order of the median of expression ranks in 83 transcriptome datasets. **Table S2.** The two nucleotide sequences confirmed to have promoter activities in this study. **Table S3.** Median of ranks and expression rank of top 13 genes in Table 1 evaluated from 83, 75 and 8 transcriptome datasets. **Table S4.** Primers for construction of transformants.

**Additional file 2: Figure S1.** Construction of transformants for promoter activity test.** Figure S2.** Fungal cell weight after 10 days in V8 and 20 days in PDB liquid medium culture.

## Data Availability

The gene list in order of the median of expression ranks in 83 transcriptome datasets is provided as the supplementary data of Additional file 1: Table S1. The procedure of constructing the transformants were described in Additional file 2: Figures S1 and S2, together with the primer list in Additional file 1: Table S3. The in-house transcriptome data is available in NCBI Gene Expression Omnibus (acc. no. GSE136041).

## Abbreviations

GUS: β-Glucuronidase
LC–MS/MS: Liquid chromatography − tandem mass spectrometry
PDB: Potato dextrose broth
RiPP: Ribosomally synthetized and post-translationally modified peptide
SMB: Secondary metabolite biosynthesis
UTR: Untranslated region
V8: V8 vegetable juice
