# Supplementary material for: Promoter tools for further development of Aspergillus oryzae as a platform for fungal secondary metabolite production
Source: Fungal Biol Biotechnol. 2020 Mar 23;7:3. doi: 10.1186/s40694-020-00093-1 (PMC7092444; doi:10.1186/s40694-020-00093-1)
Supplement: Supplementary file 2 — Additional file 2: Figure S1. Construction of transformants for promoter activity test. Figure S2. Fungal cell weight after 10 days in V8 and 20 days in PDB liquid medium culture. [file 40694_2020_93_MOESM2_ESM.pptx]

## Slide 1
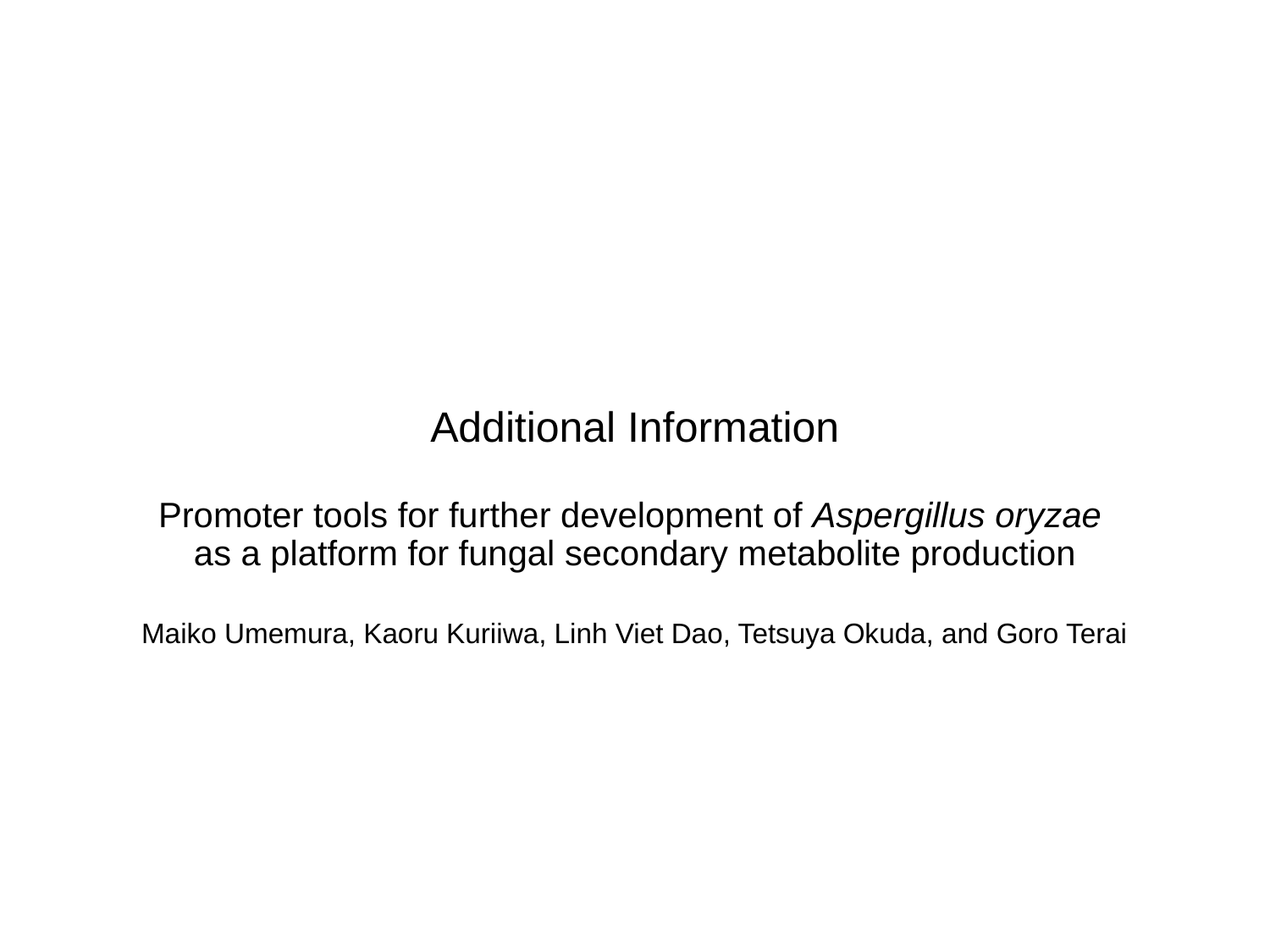

Additional Information
Promoter tools for further development of Aspergillus oryzae
as a platform for fungal secondary metabolite production
Maiko Umemura, Kaoru Kuriiwa, Linh Viet Dao, Tetsuya Okuda, and Goro Terai

## Slide 2
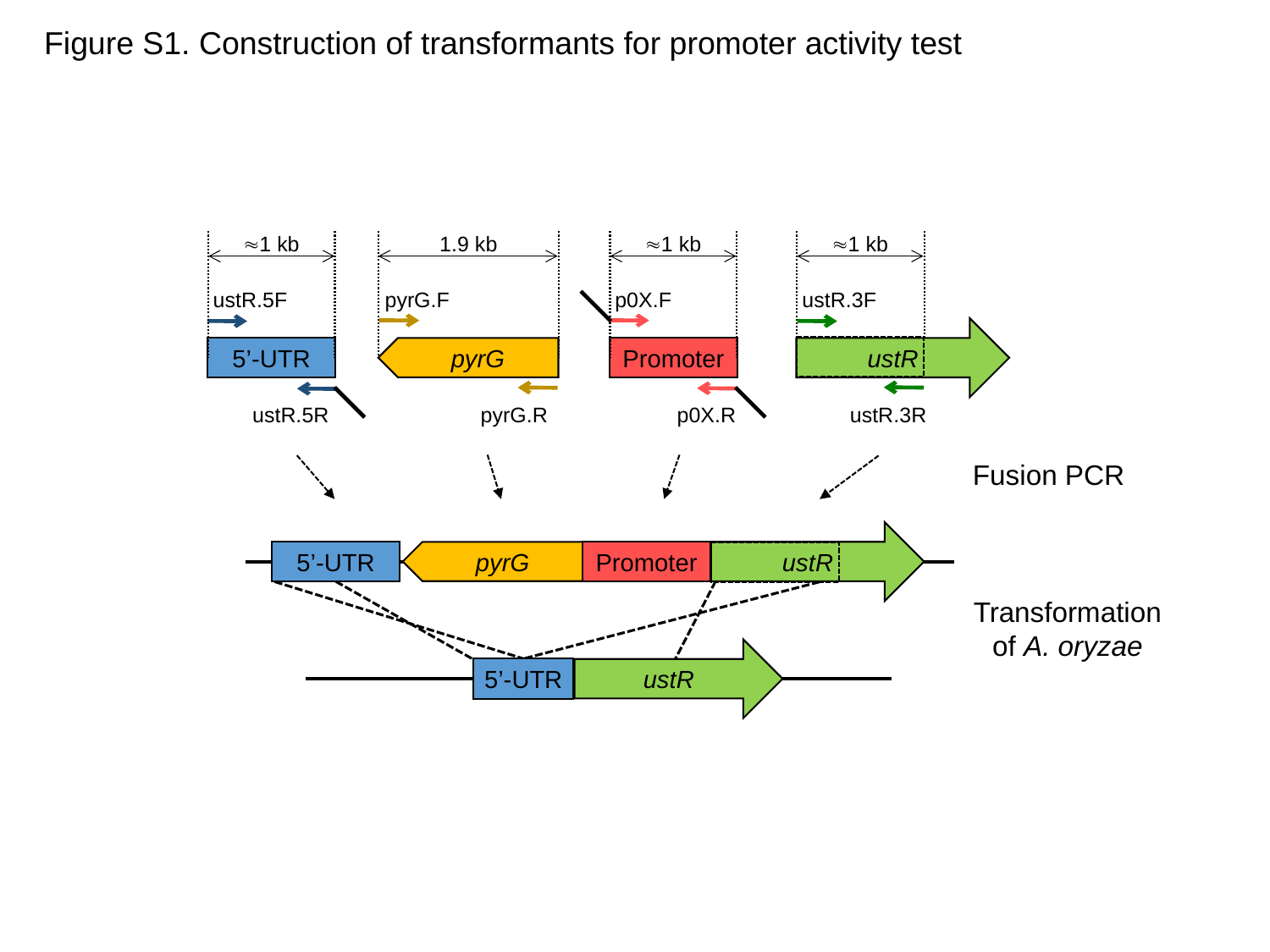

Figure S1. Construction of transformants for promoter activity test
1 kb
1.9 kb
1 kb
1 kb
ustR.5F
pyrG.F
p0X.F
ustR.3F
ustR
5’-UTR
Promoter
pyrG
ustR.5R
pyrG.R
p0X.R
ustR.3R
Fusion PCR
ustR
5’-UTR
Promoter
pyrG
Transformation
of A. oryzae
ustR
5’-UTR

## Slide 3
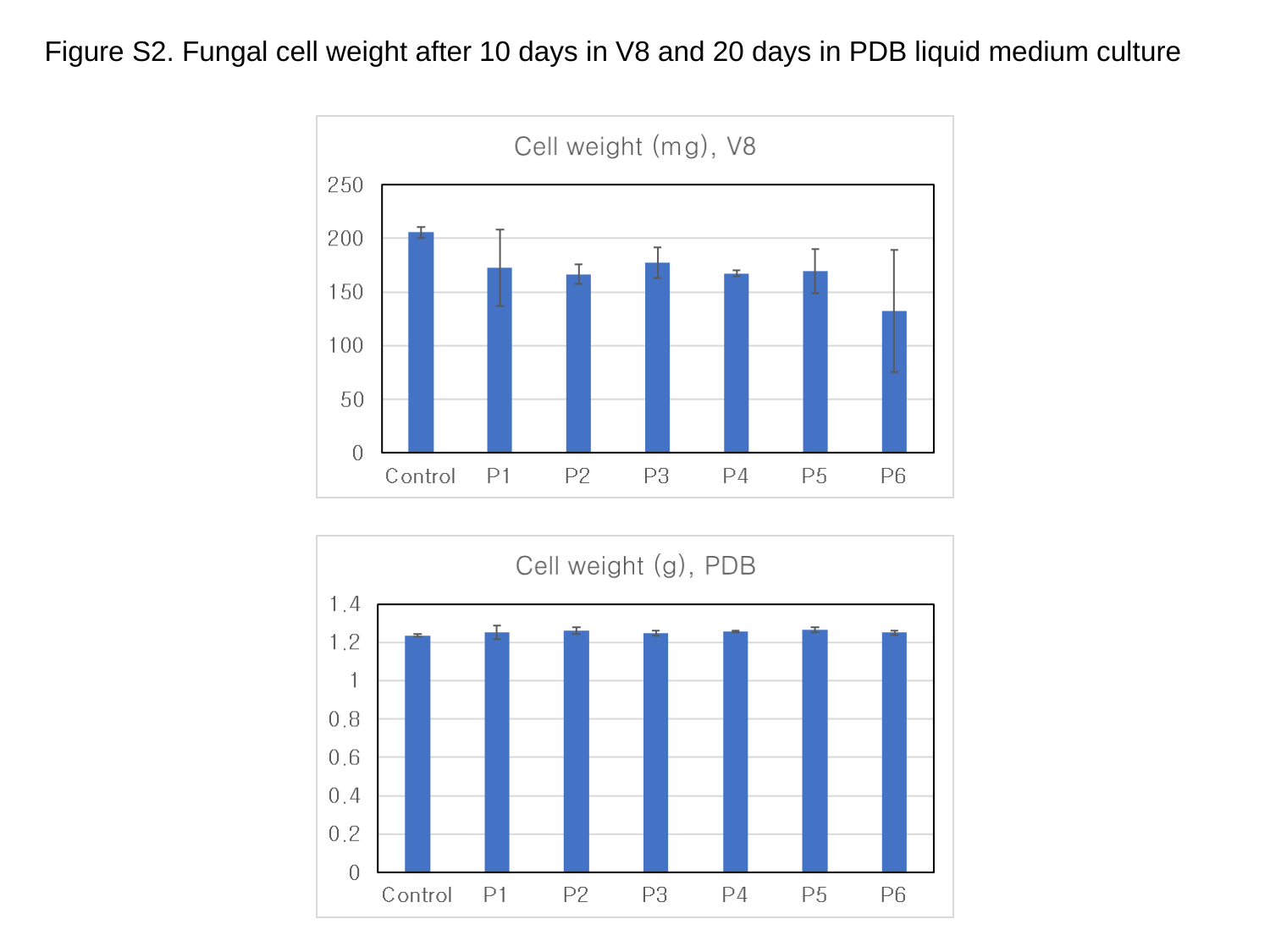

Figure S2. Fungal cell weight after 10 days in V8 and 20 days in PDB liquid medium culture
